# The United Kingdom Childhood Cancer Study of exposure to domestic sources of ionising radiation: 2: gamma radiation

**DOI:** 10.1038/sj.bjc.6600277

**Published:** 2002-06-05

**Authors:** UK Childhood Cancer Study Investigators

**Affiliations:** UKCCS, University of Leeds, Institute of Epidemiology, 30 Hyde Terrace, Leeds LS2 9LN, UK

**Keywords:** childhood cancer, gamma dose rate, radon interactions, acute lymphoblastic leukaemia, non-Hodgkin's lymphoma, central nervous system tumours

## Abstract

This article reports measurements of household levels of gamma and cosmic rays at the addresses of children with cancer at the time of diagnosis and six months before, and of similar data at the addresses of control children. There is no indication of increased risk with increasing dose rates either in matched or unmatched analyses, with or without adjustment for deprivation. Sub-division by diagnostic group did not reveal any association with any specific types of malignancy. Studies of the relationship between household gamma rays and radon concentration show no evidence of any interactions.

*British Journal of Cancer* (2002) **86**, 1727–1731. doi:10.1038/sj.bjc.6600277 www.bjcancer.com

© 2002 Cancer Research UK

## 

The United Kingdom Childhood Cancer Study had, as one of its *a priori* hypotheses, that ionising radiation from natural sources within households might cause childhood cancer. To address this question separate household measurements were made of radon gas concentration and of penetrating external radiation (predominantly from terrestrial gamma rays and cosmic rays). Whilst there was special interest in the biological effects of high linear energy transfer (LET) irradiation from radon and its short term decay products (UKCCS Investigators, 2002), there was little reason to think that there would be detectable haematological effects arising from differential exposures to domestic gamma ray radiation. Risk estimates, based on standard radiological protection approaches, have suggested that up to about 20% of childhood and young adult leukaemias (aged 0–24) in the UK could be due to natural low-LET radiation, including terrestrial gamma rays (COMARE, 1996; Simmonds *et al*, 1995). However, variation in gamma ray levels between households are likely to be quite modest. There are few published studies of possible childhood cancer risks from domestic gamma ray levels. One correlation study has suggested that there may be a negative trend in risk for childhood leukaemia associated with domestic gamma levels (Muirhead *et al*, 1992). This trend was partially reversed when administrative district data were analysed with adjustment for counties. However, a subsequent study, which also adjusted for area socio-economic status, showed no association (Richardson *et al*, 1995).

There have been many studies of terrestrial and cosmic gamma ray from many countries recording natural (and local) variation in dose rate (UNSCEAR, 1993). The UK study of this type (Wrixon *et al*, 1988), conducted by the National Radiological Protection Board (NRPB) with data on over 2000 households from measurements taken in the 1980's, allows comparisons with the results of the present study.

## METHODS

**Figure 1:**
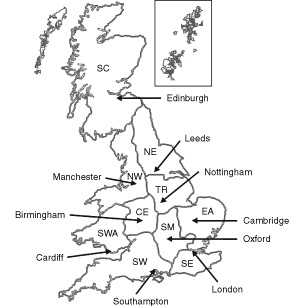
Regions and study centres of the UKCCS. UKCCS study regions: SC Scotland; NE North-east; NW North-west; TR Trent; CE Central; EA East Anglia; SM South Midlands; SWA South-Wales; SW South-west; SE South-east

The UKCCS was designed as a population-based matched case–control study covering the whole of Great Britain. Ten regional centres (Figure 1

) administered the same study protocol, with minor regional modification to satisfy local ethical committee approval and practical considerations. The study methods are given in more detail elsewhere (UKCCS Investigators, 2000).

### Study subjects

During the study period, all children with malignant neoplasms aged under 15 years in England, Wales and Scotland were registered through liaison with oncologists and paediatricians. The study began accruing cases in 1991 for Scotland and in 1992 for England and Wales. Scotland terminated case registration at the end of 1994. In England and Wales, registration of solid tumours was terminated at the end of 1994, of non-Hodgkin's lymphoma in 1995, and of leukaemias in 1996. All neoplastic diagnoses were submitted for central pathological review.

With few exceptions, each case had two randomly selected controls, matched by sex, calendar month of birth and (former) Family Health Services Authority (England and Wales) or Scottish Health Board of residence. Children were eligible if they were born in Great Britain, had no prior malignancy and were not in local authority care. Subjects were ineligible if they themselves or their parents had lived outside Great Britain for the three months leading up to diagnosis. Eligible controls who declined to participate were replaced until two controls were enrolled (UKCCS Investigators, 2000).

### Measurement of gamma radiation

A face-to-face interview was conducted with each child's parents, covering social, occupational and medical histories of the child and parents. A full residential history for the child was also collected and all addresses lived in by the child for 6 months or more were targeted for measurement. A written request to participate was then made to each residence. Following agreement, two radon and two thermoluminescent dosimeters (TLD) or ‘gamma’ detectors were sent to each household with instructions to place one of each in the main bedroom and in the main living area. After 6 months had elapsed a letter was sent recalling the detectors, which were returned to the NRPB for processing and measurement of the cumulative exposure. This study reports on the results of the address of the case at diagnosis, that is, those who had lived at that address for at least the previous 6 months.

The system used for passive environmental photon monitoring consisted of a modification of a standard body TLD used for personal photon monitoring and the assessment of dose is based on two 30% LiF;Mg;Ti detector elements. These detectors respond to all types of penetrating ionising radiation including terrestrial gamma rays and secondary particles resulting from cosmic rays.

The results are expressed as absorbed dose to air. Annual estimates are in micro gray (μGy) per year and hourly dose rates in nano gray (nGy) per hour. The results given in this study include the dose from cosmic rays; this varies with height above sea level, but differences within the UK are small. The mean has been estimated to be 32 nGy per hour (UNSCEAR, 2000) or 280 μGy per year.

The main problems encountered with the detectors were occasional damaged detectors, faulty readouts, and differences between the recorded doses on the two detectors of greater than 20%. In all these cases the result was excluded from the analysis. It is estimated that this occurred for between 5 and 10% of measurements. Where the readings of the two detectors differed by less than 20%, the mean value was used. Doses accumulated away from the household of measurement were allowed for by subtracting 2.3 μGy for each day in storage, and 0.9 μGy for each day in transit.

Detectors were intended to be in place for 6 months but the period over which measurements were actually made varied. The analyses were restricted to the measurements of the doses that had accumulated with the detectors in place within a household for both living-room and bedroom for between 5 and 7 months. Over 99% of all detectors met this requirement.

### Deprivation scores

An area-based index of deprivation for the child's residence at the time of diagnosis, and 6 months before, based on an index used in a previous study of childhood leukaemia (Draper *et al*, 1991), was used to provide a proxy socio-economic adjustment (UKCCS, 2000). The index was calculated for the smallest 1991 census area, that is an enumeration district (ED) in England and Wales or an output area (OA) in Scotland. Proportions of the following variables within each ED/OA were calculated: economically active persons unemployed, households with no car, households not owner-occupied, and overcrowded households (more than one person per room). The variables were log-transformed (to reduce skewness to zero), standardised (mean of zero and standard deviation of one) and the standard deviates summed for each ED/OA. The deprivation index was used to provide seven categories, with equal numbers of ED/OA's in each category. Greater values in the deprivation index represent greater deprivation. All targeted houses were given a validated postal code and allocated to an ED or OA from the 1991 census.

### Statistical analysis

All statistical analyses were performed using Stata version 6 (Stata Corporation, 1998). All estimates of risk are presented as odds ratios, derived using conditional logistic regression modelling. To check that data for incomplete sets, dropped after matching, did not unduly influence the results, all analyses were repeated with logistic regression modelling, adjusted for age and sex (the matching variables). The data were checked for confounding between socio-economic status and household gamma.

## RESULTS

The parents of 3838 children with cancer and 7629 children without cancer were interviewed, representing 87% of eligible cases and 64% of eligible controls. Following interview, measurements were obtained from the home at diagnosis of 2165 cases and 5086 controls. Nearly all (97%) estimates were based on readings obtained from both the bedroom and the living room.

The case household participation for TLD ‘gamma’ detection was virtually identical to that for radon measurements. Thus all the issues that arose from the differential response rates of the case and control families and from the overall response rates are similar to those discussed in the accompanying radon paper (UKCCS, 2002). Table 1

**Table 1:**
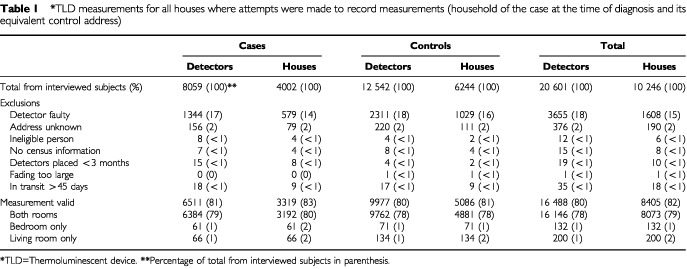
*TLD measurements for all houses where attempts were made to record measurements (household of the case at the time of diagnosis and its equivalent control address)

gives details of what happened to the detectors after they were sent to those households which agreed to participate. The study accrued results from over 10 000 households. Figure 2

**Figure 2:**
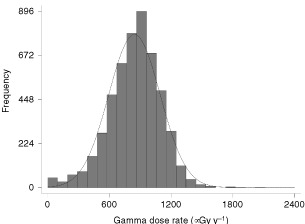
Frequency of absorbed dose rate in control houses.

shows the distribution of gamma rays results from the 5086 control households, showing a mean annual absorbed dose of 843 μGy with individual values varying from undetectable levels (i.e. under 100 μGy y^−1^) to a maximum of 2027 μGy y^−1^.

Table 2

**Table 2:**
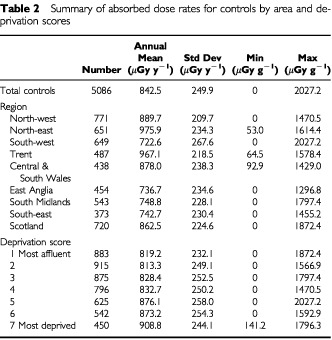
Summary of absorbed dose rates for controls by area and deprivation scores

gives the control measurements reported for nine regions (two, Central and South Wales being combined) and for the seven socio-economic groups. The mean household dose rates vary throughout the country: the highest levels being in the North East Region of England and Trent and the lowest in the South West Region of England (See Figure 1). This is commented on in the discussion. The mean dose rates vary with deprivation score, the most deprived households having the highest mean dose rate. Although there are trends in the dose rates by deprivation, and there is also an apparent geographical variation, neither set of results is statistically significant due to the wide scatter of results and the consequentially high standard deviations.

Studies of dose rate by measurement year and months by region showed little variation over the period of the study (results not shown). Table 3

**Table 3:**
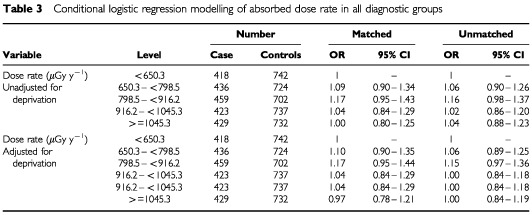
Logistic regression modelling of absorbed dose rate in all diagnostic groups

shows the overall odds ratio (for any cancer) and household dose rate based on quintiles, using both matched and unmatched analyses. The results show no relationship between either crude results or results adjusted for deprivation and increased odds ratios for childhood cancer. Separate data from six diagnostic groups are given in Table 4

**Table 4:**
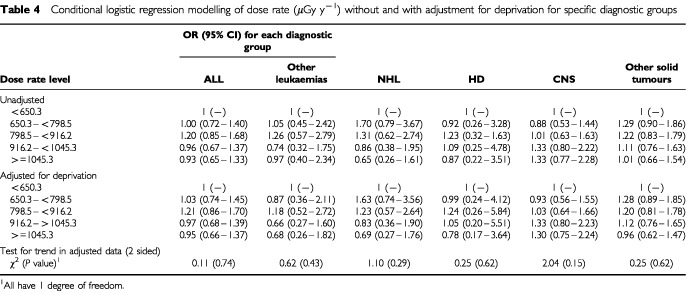
Conditional logistic regression modelling of dose rate (μGy y^−1^) without and with adjustment for deprivation for specific diagnostic groups

. There is no suggestion of either an increasing or decreasing risk from any of the groups. For CNS tumours, the odds ratios increase with increasing dose rate only to a maximum of 33% and the trend is not statistically significant (*P*=0.15)

### Radon* –* Gamma interaction

The relationships between TLD-derived doses divided into thirds (under 758.1, 758.1–956.6, over 956.6) and radon concentrations split into the five predefined levels (see radon paper, this issue) of 0–24, 25–49, 50–99, 100–199 and 200+ Bqm^−3^ is shown in Table 5

**Table 5:**
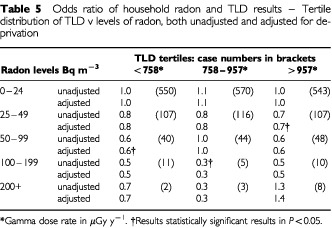
Odds ratio of household radon and TLD results – Tertile distribution of TLD v levels of radon, both unadjusted and adjusted for deprivation

. There is no suggestion of any interactions, apart from a weak association at the highest gamma and radon level, which is not statistically significant. When the analyses are conducted using quintiles of radon exposure, no such association appears.

## DISCUSSION

To our knowledge this is the first case–control study directly measuring domestic gamma ray and cosmic ray levels and relating this to the risk of all childhood cancers. The results relate to the households of the affected children at the time of diagnosis, and are limited to children who had lived at the address for a minimum period of 6 months, the control households having the same limitations. The study is limited by accuracy of the TLD measurements, including inherent detector variability, storage, transport, householder compliance and the assumption of constant cosmic ray dose rate irrespective of altitude of the household. Some indication of combined uncertainties can be obtained from Figure 2. If it is assumed that the dose from cosmic rays is approximately consistent at 34 nGy per hour, then this should produce a reading on all detectors of about 300 μGy per year. The fact that there were some TLD measurements in the below 300 μGy per year category (see Figure 2) suggests that these measurements can carry uncertainties of at least 400 μGy per year, or about 50% of the mean value of the distribution.

Cosmic ray dose rates increase systematically with increasing altitude. However, most of the population live at relatively low altitudes (see below), so it is reasonable to assume the variation in dose rates seen in the analysis is mostly due to terrestrial gamma rays.

The geographical trends in gamma-ray dose rate results by Region that might be expected from knowledge of geological variations are obscured by variation in population density. For example: studies of Wrixon *et al* (1988) have shown that the environmentally based geographical weighted (outdoor) gamma ray dose rates for Devon and Cornwall are the highest in the UK. However the population weighted (indoor) gamma value for Devon is around the median for county values in the UK. This is because most of the population live in cities and towns such as Plymouth, Exeter and Torbay which are not particularly high gamma ray areas – the population of Dartmoor, a high gamma ray area, is quite low. This is compounded by the grouping into health regions – the Southwest grouping includes, for example, Hampshire with some 600 000 homes, whilst Cornwall has only 170 000 homes. If data from Wrixon *et al* (1988) on indoor gamma dose rates are amalgamated (as in the present study), a similar ranking would be achieved.

Terrestrial gamma rays have been estimated to contribute about 30% of the total natural annual low-LET dose to bone marrow, most of the remainder being due to cosmic rays (∼40%) and natural internal radio nuclides with the body (∼30%) (Simmonds *et al*, 1995). If 20% of young persons' leukaemias are related to natural low-LET radiation, as has been suggested from standard radiological risk estimates for ages 0–24 (Simmonds *et al*, 1995; COMARE, 1996), then about 6% of these leukaemias may be attributable to terrestrial gamma rays. It is unlikely that a relative risk of this magnitude would be detectable in the present study after its statistical power, modest variations in dose rate and limitations of data collection are taken into account. Thus, the results of the study are reassuring, in that they do not indicate any measurable risk from natural gamma rays in the UK. Nevertheless, they do not contradict expectations of a small risk based on standard methods of risk assessment.

The results do not indicate either a positive or negative trend between gamma ray exposure and childhood cancer, the only exception being CNS tumours which show a weak (statistically not significant) positive trend. That one such trend should be observed when six groups are examined is hardly surprising. When the estimated mean cosmic ray dose rate is taken into account, the mean household terrestrial gamma-ray dose rate of 62.1 nGy h^−1^ is very similar to that found in the NRPB material (Wrixon *et al*, 1988) of 60.1 nGy h^−1^.

The increase in measured household exposure with increase in the deprivation index necessitated adjustment for socio-economic factors, but this makes no significant difference to the results. With the exception of the highest measured gamma-ray dose rate and radon concentration there was no evidence of any association between the gamma-ray and the results of the radon analysis. The number of households for which the highest levels of both were recorded (8) was, however, very small.

In conclusion, in line with standard risk estimates, the findings from this study are broadly reassuring.

### List of investigators

#### Writing Committee:

RA Cartwright, G Law, E Roman, E Gilman, OB Eden, M Mott , K Muir, D Goodhead, G Kendall.

#### Management Committee:

KK Cheng, Central Region; N Day, East Anglia Region; RA Cartwright, A Craft, North East Region; JM Birch, OB Eden, North West Region; PA McKinney, Scotland; J Peto, South East Region; V Beral, E Roman, South Midlands Region; P Elwood, South Wales Region; FE Alexander, South West Region; CED Chilvers, Trent Region; R Doll, Epidemiological Studies Unit, University of Oxford; GM Taylor, Immunogenetics Laboratory, University of Manchester, Manchester; M Greaves, Leukaemia Research Fund Centre, Institute of Cancer Research; DT Goodhead, Medical Research Council, Radiation and Genome Stability Unit, Harwell; FA Fry, National Radiological Protection Board; G Adams, UK Co-ordinating Committee for Cancer Research.

#### Regional investigators:

KK Cheng, E Gilman, Central Region; N Day, J Skinner, D Williams, East Anglia Region; RA Cartwright, A Craft, North East Region; JM Birch, OB Eden, North West Region; PA McKinney, Scotland; J Deacon, J Peto, South East Region; V Beral, E Roman, South Midlands Region; P Elwood, South Wales Region; FE Alexander, M Mott, South West Region; CED Chilvers, K Muir, Trent Region.

#### Leukaemia Research Fund Data Management Processing Group:

RA Cartwright, G Law, J Simpson, E Roman.

A complete list of investigators is given in: The United Kingdom Childhood Cancer Study: objectives, materials, and methods. *Br J Cancer* (2000) **82:** 1073–1102
